# Analysis of the health product pipeline for poverty-related and neglected diseases using the Portfolio-to-Impact (P2I) modeling tool

**DOI:** 10.12688/f1000research.24015.1

**Published:** 2020-05-21

**Authors:** Shashika Bandara, Nick Chapman, Vipul Chowdhary, Anna Doubell, Amelia Hynen, George Rugarabamu, Alexander Gunn, Gavin Yamey

**Affiliations:** 1 Center for Policy Impact in Global Health, Duke Global Health Institute, Duke University, Durham, NC, 27708, USA; 2 Policy Cures Research, Sydney, NSW, 2010, Australia

**Keywords:** neglected diseases, product development, emerging infections, research and development, research funding

## Abstract

**Background: **To estimate how much additional funding is needed for poverty-related and neglected disease (PRND) product development and to target new resources effectively, policymakers need updated information on the development pipeline and estimated costs to fill pipeline gaps.

**Methods: **We previously conducted a pipeline review to identify candidates for 35 neglected diseases as of August 31, 2017 (“2017 pipeline”). We used the Portfolio-to-Impact (P2I) tool to estimate costs to move these candidates through the pipeline, likely launches, and additional costs to develop “missing products.” We repeated this analysis, reviewing the pipeline to August 31, 2019 to get a time trend. We made a direct comparison based on the same 35 diseases (“2019 direct comparison pipeline”), then a comparison based on an expanded list of 45 diseases (“2019 complete pipeline”).

**Results:** In the 2017 pipeline, 538 product candidates met inclusion criteria for input into the model; it would cost $16.3 billion (B) to move these through the pipeline, yielding 128 launches. In the 2019 direct comparison pipeline, we identified 690 candidates, an increase of 152 candidates from 2017; the largest increase was for Ebola.  The direct comparison 2019 pipeline yields 196 launches, costing $19.9B. In the 2019 complete pipeline, there were 754 candidates, an increase of 216 candidates from 2017, of which 152 reflected pipeline changes and 64 reflected changes in scope. The complete pipeline 2019 yields 207 launches, costing $21.0B. There would still be 16 “missing products” based on the complete 2019 pipeline; it would cost $5.5B-$14.2B (depending on product complexity) to develop these products.

**Conclusion: **The PRNDs product development pipeline has grown by over a quarter in two years. The number of expected new product launches based on the 2019 pipeline increased by half compared to 2017; the cost of advancing the pipeline increased by a quarter.

## Introduction

A growing body of evidence has shown that the ambitious global health goals established by the international health community, including the health-related Sustainable Development Goals (SDGs), are unlikely to be achieved with today’s health technologies alone ^1– 4^. New breakthrough technologies will be needed to accelerate the current rate of mortality reduction.

For example, the 2013 report of the *Lancet* Commission on Investing in Health (CIH) showed that the development and widespread deployment of new technologies would be needed to achieve a “grand convergence” in global health ^1^. The CIH defined grand convergence as a universal reduction in deaths from infections and maternal and child health conditions down to the mortality levels achieved in 2011 by the best-performing middle-income countries (e.g. China and Chile) ^1^. Unfortunately, progress on mortality from several conditions has been slow since this report was published. If the global trends in maternal mortality and mortality from tuberculosis (TB) achieved in 2010–16 were to continue, the convergence targets would not be achieved until 2067 for maternal mortality and 2074 for TB mortality, respectively ^4 ^. This slow progress underscores the need for new medicines, vaccines, diagnostics, and other disease control tools.

There are several signs suggesting that policymakers at global and national levels do recognize the importance of funding product development for poverty-related and neglected diseases. First, funding levels for health research and development (R&D) were included in the indicators that are linked to the SDG health targets. For example, SDG Indicator 3.b.2 includes “official development assistance (ODA) for medical research and basic health sectors as a % of gross national income (GNI) and as a % of all ODA, by donor country.” SDG Indicator 9.5.1 is “gross domestic R&D expenditure on health (health GERD) as a % of gross domestic product (GDP).” Second, the new Global Action Plan for Healthy Lives and Wellbeing for All, which commits 12 multilateral health and development organizations to improving their collaboration on achieving the SDGs, includes an effort to scale up health R&D. Third, several new initiatives have been launched in recent years to mobilize additional funding for global health product development, such as the Global Health Innovative Technology Fund and the Coalition for Epidemic Preparedness Innovations ^5^. Fourth, a number of new global health R&D coordination mechanisms have also been launched, such as the Global Antimicrobial Resistance R&D Hub and the World Health Organization’s Global Observatory on Health R&D ^5^.

To estimate how much additional funding is needed for global health product development and to target any new resources effectively, policymakers need up-to-date information on the status of the development pipeline and the estimated costs to fill gaps in the pipeline. In particular, as we have previously argued, they need information on “which candidates are currently in the pipeline and at what development phase; the estimated costs to accelerate this portfolio of candidates to production; the anticipated product launches that would result from such acceleration; and the critical, highly needed products that would still be “missing” under the status quo.” ^6^

In order to generate such information, in 2018 we published the first ever study to use a new financial modeling tool, called the Portfolio-to-Impact (P2I) tool, that can be used to estimate the costs of moving a portfolio of candidates along the pipeline ^6^. In that 2018 study, we first conducted a comprehensive, rigorous pipeline portfolio review to identify product candidates for 35 neglected diseases as of August 31, 2017. We found 538 candidates that met inclusion criteria for input into the modeling tool. Using this tool, we estimated that it would cost about $16.3 billion (range $13.4B-19.8B) to move these 538 candidates through the pipeline, with three-quarters of the costs incurred in the first 5 years, resulting in about 128 (89-160) expected product launches by 2029. 

Among these launches, our modeling suggested that there would be few launches of complex new chemical entities (NCEs). We also found that, based on the pipeline as of August 31, 2017, launches of highly efficacious HIV, TB, or malaria vaccines would be unlikely. We estimated that the additional costs to launch one of each of 18 priority “missing” products would be $13.6B, assuming lowest product complexity or $21.8B assuming highest complexity ($8.1B-36.6B).

In this current study, we have conducted a new pipeline portfolio review and we have updated the cost modeling. There are several reasons why this updated study was needed. First, the product development pipeline is never static—it changes frequently—and so we wanted to gain an understanding of how the pipeline has changed. We conducted a new pipeline review up to August 31, 2019, i.e. exactly two years on from our last study, allowing us to show how much change can be expected over such a two-year period. Second, we wanted to assess whether the prospects have improved for launching critically needed products, such as vaccines for HIV, TB, and malaria. Third, our new study was timely given the launch of a new target product profile (TPP) directory developed by the Special Programme for Research and Training in Tropical Diseases (TDR). We hoped that we could potentially compare our new findings on product gaps with the gaps identified in the new TPP directory. Finally, we wanted the new findings to help identify the most promising ways in which the pipeline can be better managed to accelerate the development and deployment of critically needed health technologies for poverty-related and neglected diseases.

## Methods

In this section, we begin, in sub-section (i), by summarizing the development of the P2I tool. While the tool development has previously been published in detail ^6, 7^, we wanted to summarize the key steps so that readers could understand the underlying principles of—and the assumptions and data sources underlying—the tool. In sub-section (ii), we describe how we conducted the pipeline portfolio review and in (iii) we explain how we classified each candidate into its product type (which we call “archetype”, e.g. simple NCE, complex NCE). In sub-section (iv) we describe how we inputted the pipeline of candidates into the P2I model. In sub-section (v), we explain how we estimated the costs of priority “missing” products. Finally, in sub-section (vi), we describe our sensitivity analysis.

### (i) Summary of how the P2I.v2 model was developed

**Figure 1.**
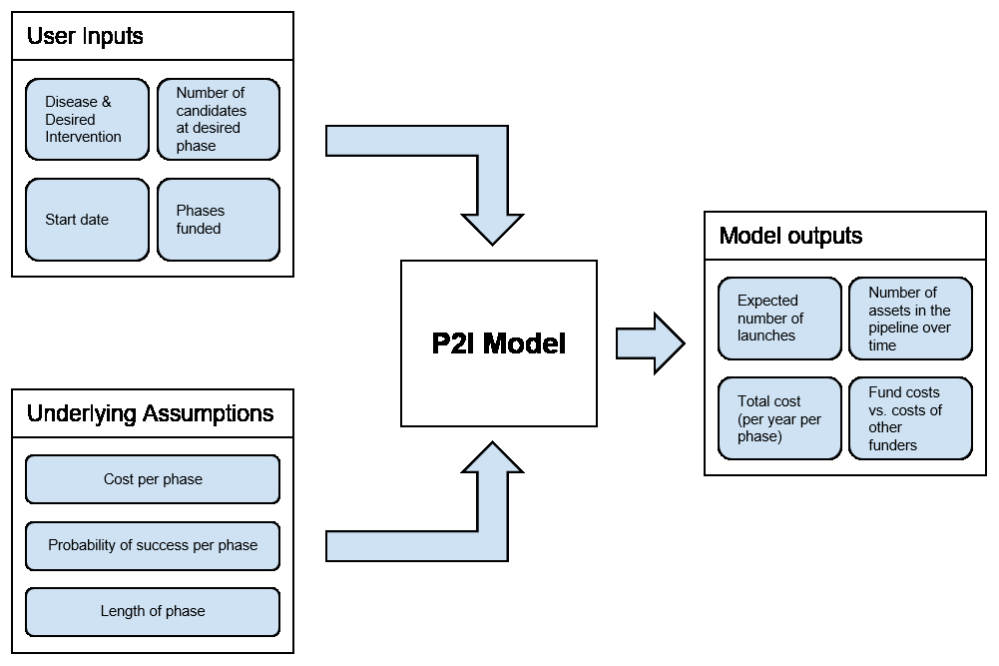
Conceptual overview of the P2I model. This figure is taken with permission from Young R, Bekele T, Gunn A *et al.* Developing new health technologies for neglected diseases: a pipeline portfolio review and cost model. Gates Open Research 2018, 2:23.

**Figure 2.**
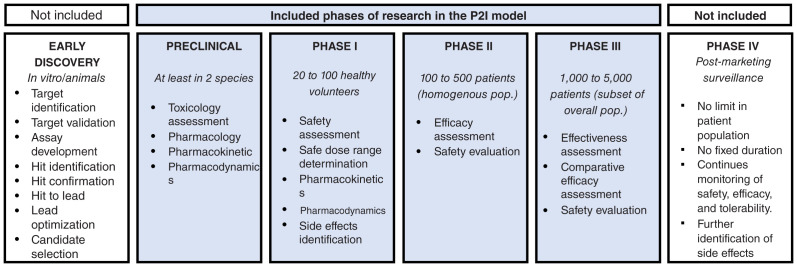
Development phases included in the P2I model. This figure has been adapted with permission from Health Product Research and Development Fund: A Proposal for Financing and Operation. WHO/TDR, 2016.

The P2I model is a deterministic modeling tool that enables users to estimate funding needs to move a portfolio of candidate health products through the pipeline from advanced preclinical to phase III clinical trials, as well as potential product launches over time. The first version of the P2I tool, called P2I version 1 (P2I.v1) is a Microsoft Excel-based tool that is freely available online ^7^. In the first study to apply this tool to the pipeline of candidate products for neglected diseases, we made a number of adaptations to P2I.v1, and called the adapted version P2I version 2 (P2I.v2); this adapted version is also an open access Microsoft Excel-based tool ^6^. Both the P2I.v1 and P2I.v2 models are based on assumptions for costs, attrition rates (probability of success), and cycle times per phase for four phases of research—advanced preclinical to phase III—for different product types (archetypes). Figure 1 shows a conceptual overview of the P2I model, and Figure 2 shows which phases are included.

The P2I.v1 model has 11 archetypes: simple or complex vaccines; simple or complex NCEs; simple, innovative, or complex repurposed drugs; simple or complex biologics; and two diagnostic archetypes—assay development or simple technical platform development. We made a number of adaptations in the development of P2I.v2, including the addition of “unprecedented vaccines” (for HIV, TB, and malaria) and vector control products. Table 1 describes the 14 archetypes in the P2I.v2 model, which is the version we have used in this new study.

**Table 1.** Descriptions and examples of the 14 archetypes included in the P2I.v2 model. Adapted from Young *et al*. (2018) ^6^ under the terms of the Creative Commons Attribution 4.0 International license (CC-BY 4.0).

Product archetype		Description	Examples	Further definition
**Repurposed** **drug**	Simple	Drug has sufficient safety data to start development in phase II	azithromycin, doxycycline	Any drug that has already been approved for market use in humans and is now being used in a new formulation or for a new indication.
	Complex	Drug requires some phase I clinical trials to verify safety in humans	moxidectin	Any drug that has already been approved for market use in humans and still requires clinical trials to verify safety in humans.
**New chemical** **entity (NCE)**	Simple	Validated target/ mechanism of action	primaquine	Any chemically synthesized drug that is part of a well-established class of drugs or has a mechanism of action that has already been approved for market use in humans. It is not the first of its class to be approved.
	Simple - TB	Validated target/ mechanism of action	Sutezolid (PNU-100480)	Any candidate that meets criteria for a simple NCE and is developed for TB.
	Complex	Novel target/ mechanism of action without understanding of disease pathogenesis	imatinib	Any chemically synthesized drug that is first in its class as determined by having a novel target/mechanism of action regardless of the drug’s indication.
**Biologics**	Simple	Validated target/ mechanism of action	human monoclonal antibody m102.4	Any drug that is synthesized from a living organism that is part of a well- established class of drugs or has a mechanism of action that has already been approved for market use in humans. It is not the first of its class to be approved.
	Simple - TB	Validated target/ mechanism of action	CPZEN-45	Any candidate that meets criteria for a simple biologic and is developed for TB.
	Complex	Novel target/ mechanism of action	polyclonal IgG antibodies	Any drug that is synthesized from a living organism that is first in its class as determined by having a novel target/ mechanism of action regardless of the drug’s indication.
**Vaccines**	Simple	Platform has been used to develop other vaccines.	Hep A, Hep B, polio, killed or live attenuated vaccines.	Any vaccine platform that has been extensively researched and approved for use in the past. Conferral of immunity against disease-causing microorganism is expected as natural immunity to the pathogen is protective. Platform is likely to elicit robust protective response.
	Complex	Requires completely novel approach; no platform; no existing research	Pneumococcal conjugate vaccine, meningitis B, DNA vaccine or mRNA vaccines	Any vaccine platform that requires a novel approach that has not been successfully approved for use in the past. Conferral of immunity against disease-causing microorganism is difficult to induce and maintain and natural immunity is not protective. Platform may elicit incomplete/ insufficient immunity and require boosting over time.
	Unprecedented	Defined in the same way as complex vaccines, but with lower probabilities of success	HIV, TB, malaria vaccines	All vaccine candidates for HIV, TB, and malaria are classified as “unprecedented”; they have much higher attrition rates at phases II and III than other complex vaccines
**Other products**		This category refers to vector control products	Long-lasting insecticide- treated bed nets, new chemical pesticides	Chemical pesticides intended for global public health use that aim to control/kill vectors associated with transmitting poverty-related diseases; biological control interventions that aim to control/ kill vectors associated with transmitting poverty-related diseases; veterinary vaccines designed to prevent animal- to-human transmission of neglected diseases.
**Diagnostics**	Assay development	Development of a diagnostic assay	Lateral flow tests, qualitative/ quantitative molecular tests	Any new diagnostic that represents menu extension on an existing platform with an assay targeting a neglected disease.
	Simple technical platform development	Development of a technical platform that enhances current technology	Ultrasensitive malaria rapid diagnostic test	Any new diagnostic that relies on a novel approach to sample processing or target detection.

For each of the 14 different archetypes in P2I.v2, the model has assumptions on costs, attrition rates, and cycle time per phase. As described in detail in Terry *et al*, assumptions on development costs for each phase of product development for the archetypes included in the P2I.v1 model were initially based on an analysis of clinical trial costs from Parexel’s R&D cost sourcebook ^7^. The assumptions on attrition rates and cycle times for each phase were initially based on the historical attrition rates and cycle times of more than 25,000 development candidates. All of the assumptions were further refined and validated based on (i) academic literature, (ii) industry publications and databases, and (iii) 133 stakeholder interviews with a wide variety of product development partnerships (PDPs), pharmaceutical companies, and major funders of global health R&D ^7^. As described in detail in reference 6, additional sources of assumptions for the new archetypes in P2I.v2, such as vector control products and unprecedented vaccines, were the McKinsey Risk-Adjusted Portfolio Model and clinical trial data shared by the Bill & Melinda Gates Foundation ^6^.

In order to use the model to analyse the pipeline of product candidates for poverty-related and neglected diseases, each candidate must first be classified into its archetype and phase, and then these candidates can be inputted into the model. There are two main model outputs. The first is “launches”; in this paper, the term launch refers to a candidate making it through phase III and thus being ready for the next steps, e.g. the regulatory and manufacturing steps. The second is the total costs to move all candidates through the pipeline from their current phase from now to 2031 (the model also gives a breakdown of these total costs into annual costs by year, from 2019 to 2031).

The model only includes candidates in advanced preclinical to phase III research, and thus the cost estimates are an under-estimate of the full costs of product development. In particular, the model excludes all costs related to basic research through lead optimization; chemistry, manufacturing, and controls; good manufacturing practice; manufacturing, build-up, and scale-up costs; regulatory or registration fees (post-phase III); and all post-market commitments (e.g., phase IV pharmacovigilance studies).

### (ii) Pipeline portfolio review

We undertook a global review of candidate products for neglected diseases, Ebola and sexual & reproductive health issues in order to capture a snapshot of the R&D pipeline as of August 31, 2019 (“2019 review”); this followed a previously published review ^6^ of the R&D pipeline as of August 31, 2017 (“2017 review”).

The scope of candidate inclusion aligned with the G-FINDER survey on global funding for R&D for neglected diseases, emerging infectious diseases and sexual & reproductive health issues, conducted by Policy Cures Research. The G-FINDER scope is based on three key principles: that the disease or health issue disproportionately affects low- and middle-income countries; that there is a need for new products (i.e. there is no existing product, or improved or additional products are needed); and that there is a market failure (i.e. there is insufficient commercial market to attract private R&D investment).

The scope of the G-FINDER project is reviewed annually in consultation with an expert Advisory Committee. As a result of these consultations, and following an initiative to survey the landscape of sexual and reproductive health R&D funding, the disease scope used for the pipeline review in 2019 was wider than in 2017, with the addition of the following diseases: chlamydia, gonorrhea, hepatitis B, HPV-cervical cancer, human T-cell lymphotropic virus-1, herpes simplex-2, mycetoma, pre-eclampsia, post-partum hemorrhage and syphilis. Snakebite envenoming was added to the G-FINDER scope after the conclusion of the 2019 pipeline review and was therefore not included in the scope of this analysis. “Reproductive health”—which included both contraception and the prevention of sexually transmitted diseases through the use of multipurpose prevention technologies (MPTs) that prevent pregnancy and sexually transmitted infections—was included as a single health issue.

Accordingly, the R&D pipeline review presented here comprises 45 individual disease or health issues and six multiple-disease groups ( *Underlying data:* Data File 1 shows the full list of diseases included in the pipeline reviews for both 2017 and 2019).

The G-FINDER scope includes drugs, microbicides, biologics, vaccines, diagnostics, vector control products, devices and MPTs. Not all product types are included for every disease or health condition: the product category is excluded if a viable commercial market is thought to exist, and additional restrictions are applied to some disease and product categories if there is potential commercial (high-income country market) overlap. For example, the G-FINDER scope excludes dengue vaccines, and HIV/AIDS drugs are included only if they are label-extensions or reformulations specifically intended for developing country use (e.g., pediatric or slow-release formulations; fixed dose combinations; low-dose formulations for prophylaxis; long-acting injectables for treatment or prophylaxis). Medical devices (except for diagnostics and contraceptives), and general or supportive therapies (e.g., oral rehydration or nutritional supplements) are not included in the scope of G-FINDER and were therefore not included in the pipeline reviews. Due to changes in the G-FINDER scope between 2017 and 2019, as described above, two disease-specific product areas were included for the first time in 2019: vaccines for leprosy, and biologics for HIV.

The pipeline review included candidates at all stages of development, from discovery through to product registration. Drugs, vaccines, and reproductive health candidate products were classified in the following five development stages: discovery; pre-clinical studies; and clinical development (Phase I, Phase II and Phase III). Diagnostics and vector control products follow different product development and regulatory pathways, so these candidate products were classified under the following stages: concept and research; feasibility and planning; design and development; and clinical validation and launch readiness. Candidates were no longer considered to be part of the R&D pipeline – and therefore were excluded from this analysis – once granted regulatory approval by a stringent regulatory authority, or if their development had been placed on hold indefinitely. Candidates in the discovery phase were also excluded, as only candidates undergoing advanced pre-clinical development up to Phase III are included in the P2I.v2 model.

Further details on the diseases and product areas within the scope of the pipeline analysis are provided in the G-FINDER R&D matrices and scope documents.

We reviewed and cross-referenced all major sources of available data on the R&D pipeline within the scope outlined above. Sources included: the G-FINDER R&D funding database; the World Health Organization “rainbow tables”; publications of the WHO Initiative for Vaccine Research (IVR) which included background documents and meeting reports and presentations prepared for the WHO Product Development for Vaccines Advisory Committee (PDVAC) and Strategic Advisory Group of Experts (SAGE) on immunizations; reports of the WHO Vector Control Advisory Group (VCAG) reports; WHO R&D roadmaps; the WHO vaccine pipeline tracker; WHO prequalification lists; multiple Unitaid landscape and technical reports; multiple clinical trial portals including ClinicalTrials.gov, the AIDS *info* clinical trial database, the Chinese Clinical Trial Registry, the Clinical Trials Registry – India and the WHO International Clinical Trials Registry Platform; disease-specific pipeline updates by the HIV Vaccines Trials Network and the Treatment Action Group; publicly available company and product development partnership R&D portfolios; journal publications; conference and meeting proceedings; and university, government, and non-profit organization websites.

Candidates were only included if an authoritative source could confirm they were in active development. The following sources were considered to be authoritative:

▪ the website of the candidate developer, if recently updated
▪ recent reports or other materials from international organizations such as WHO and Unitaid
▪ clinical trial portals
▪ correspondence with product developers
▪ correspondence with experts in the field, including FIND; the Innovative Vector Control Consortium (IVCC); the International AIDS Vaccine Initiative (IAVI); Netherlands Leprosy Relief; Program for Appropriate Technology in Health (PATH); the Sabin Vaccine Institute; and the US National Institute of Allergy and Infectious Diseases (NIAID).

### (iii) Classification of candidates into archetypes

In the pipeline portfolio review, we identified a total of 1160 product candidates for neglected diseases, Ebola and sexual & reproductive health as of August 31, 2019. We excluded 406 of these from the model because (a) they were already marketed, or in a development phase that is excluded from the P2I.v2 model (n=230), (b) there was insufficient information about the candidate from an authoritative source to be able to confirm its development phase or classify it into an archetype (n=10), or (c) the candidate was no longer in development (n=166). After these exclusions, 754 candidates were included in the model.

We then classified each of the included candidates into six primary archetypes: repurposed drugs, NCEs, vaccines, biologics, diagnostics, and ‘other products’ (which included only vector control products). Candidates classified as repurposed drugs, NCEs, or biologics were further classified as either simple or complex (with a further distinction for simple NCEs and simple biologics targeting TB); vaccine candidates were classified as either simple, complex, or unprecedented; and diagnostic candidates were classified as either assay development or simple platforms. This resulted in each candidate being assigned to one of the 14 unique archetypes outlined in Table 1.

Contraceptives, microbicides and MPTs were classified according to the constituent molecule (NCE, repurposed drug, or biologics). If there was more than one active drug ingredient in the MPT, the candidate was classified according to the most complicated component; we did not consider if the polymer or technology itself was innovative. TB drug candidates beyond Phase IIa were listed as regimens instead of individual compounds because TB treatment is regimen-based rather than monotherapy. Eight diagnostics candidates that were classified in the 2017 review as simple platforms were reclassified as assay development, reflecting the fact that ongoing activity was restricted to disease-specific assay development based on a previously validated proprietary diagnostic test technology.

The evidence used to support the classification of candidates into archetypes came from a wide range of sources, including academic literature (both original research and review articles); relevant publicly available product databases such as NCATS Inxight: Drugs; information from international clinical trials registries, including ClinicalTrials.gov and the WHO International Clinical Trials Registry Platform; websites of PDPs; patent databases; and relevant reports and news releases from bilateral and multilateral funding agencies, companies, PDPs, other product developers, and non-government organizations. In assigning each candidate product to an archetype, we documented any relevant source material that guided the classification, such as a peer-reviewed research article on the candidate’s mechanism of action. Regimens were classified according to the complexity of the most complicated component.

### (iv) Inputting the pipeline of candidates into the adapted P2I model

Following the pipeline portfolio review and classification of product candidates into their archetypes, the candidates that met our inclusion criteria were inputted into P2I.v2. For each disease and archetype, we entered the number of candidates that were in each phase of development at the time of our review. For the candidates classified as repurposed drugs, NCEs, biologics, vaccines, and “other products” (vector control products), the phases were preclinical, phase I, phase II, or phase III. For diagnostic candidates, the phases were concept and research, feasibility and planning, design and development, or clinical validation and launch readiness. The analysis was conducted in November 2019, and hence we selected 2019 as the start year for the modeling.

Based on the previously established parameters for cost, attrition rate, and time per phase for each archetype, the model estimates the costs and outcomes of moving product candidates through the pipeline from their current phase. When a candidate is classified into a particular phase in the model, it is assumed that it is at the beginning of the phase, which means that the cost of moving that candidate through to the end of its current phase is included in the total estimated costs for any given product.

We did not apply a discount rate to our cost estimates. Probability of success, length of phase, cost variables, and archetype were assumed to remain constant throughout the lifecycle of the model. Only candidates that met our inclusion criteria at the time of the pipeline portfolio review were included, meaning that the analysis did not include new candidates entering the pipeline over time.

For this paper, we have chosen a conservative approach to presenting the launches—we have considered a launch to be a binary event, i.e., we have rounded down (in this case, 2.7 rounds down to 2 launches). However, in *Underlying data:* Data File 2, we also present the results without any rounding (e.g., in this example, 2.7 launches) and with rounding to the nearest integer (2.7 would round to 3). Both of these other approaches give less conservative estimates of the number of launches. Where rounding was used, it was only applied at the very end of the model. For example, for disease X, if there were 3 simple vaccine candidates at Phase II that led to 1.3 expected launches and 3 simple vaccine candidates at Phase III that led to 1.4 expected launches, we rounded the cumulative total (in this case 2.7).

In this paper, we call the current pipeline of candidates the “2019 pipeline” (i.e. the pipeline of candidates as of August 31, 2019). We call the previously identified pipeline, published by Young et al, the “2017 pipeline” (i.e. the pipeline as of August 31, 2017) ^6^. As described below, we conducted two distinct analyses. First, we did an analysis to obtain a time trend—that is, we examined changes over time in the number of candidates in the pipeline, the estimated costs to move them through the pipeline, and the likely resulting launches. For this time trend analysis, we used the exact same scope of diseases—an “apples to apples” comparison (we call this the “direct comparison” between the 2017 and 2019 pipelines). We call the 2019 pipeline used for the direct comparison analysis the “2019 direct comparison pipeline.” Second, for the 2019 pipeline, we also used an expanded list of diseases. We call the pipeline used for the complete 2019 analysis the “complete 2019 pipeline.”

***The direct (“apples-to-apples”) comparison analysis between the 2017 and 2019 pipelines*.** The direct comparison analysis included product candidates in the 2019 pipeline that were identified using the *exact same* scope and search methodology used in 2017 ^6^. As described in the Results section, we found a higher number of candidates in the 2019 direct comparison pipeline than in the 2017 pipeline. Since we used the same approach to identify candidates for both the 2017 and 2019 direct comparison pipelines, the higher number of overall candidates identified in 2019 was due to “true” changes in the pipeline since 2017. Despite the overall increase, when disaggregated by disease and by archetype the pipeline showed both increases and decreases in the numbers of candidate products across different diseases/archetypes. The product candidates included in the 2019 direct comparison pipeline belonged to the exact same 35 diseases and 5 multiple disease categories as those included in the 2017 pipeline analysis ^6^.

***The complete 2019 pipeline analysis*.** The complete 2019 pipeline analysis included all candidates from the pipeline portfolio review that met our inclusion criteria based on an expanded scope of diseases (45 diseases and 6 multiple disease categories). Again, as described in the Results section, we found a higher number of candidates in the complete 2019 pipeline than in the 2017 pipeline, but in this case the increase was partly due to the expansion of the disease scope, as well as true changes in the pipeline. Additionally, eight diagnostic candidate archetype classifications were changed between the 2019 direct comparison pipeline and the complete 2019 pipeline. These were existing candidates captured in the 2017 pipeline, and so their archetype classification was left unchanged in the direct comparison analysis to allow a true apples-to-apples comparison. This classification was updated for the complete pipeline analysis to reflect the fact that ongoing development activity for these candidates was now restricted to disease-specific assay development based on a previously validated proprietary diagnostic test technology.

### (v) Estimating the costs of priority “missing” products

As described in the Results section below, the complete 2019 pipeline is unlikely to lead to launches of several critically needed technologies (e.g. a hepatitis C vaccine). To estimate the costs to develop products that are likely to be “missing” but are highly needed, we used the same methodology adopted by Young *et al*. ^6^ Young *et al*. used the list of “important” or “game changing” diagnostics, drugs, and vaccines prioritized by the CIH, which was developed through expert consensus ^1^. We examined the overlap between the CIH’s list of needed products and those that our modeling suggested would still be missing by 2031 based on the complete 2019 pipeline. For each missing product, we used the P2I.v2 model in a retrospective manner to estimate the number of additional candidates that would be needed at preclinical phase—over and above the existing candidates—to lead to one expected launch of that product, and the associated additional cost.

For example, we found 39 malaria vaccine candidates in the pipeline and the modeling suggested that these would result in 0.41 launches. Thus, to estimate the additional costs to reach one launch, we estimated the number of additional candidates needed at preclinical phase and the associated additional costs to achieve an additional 0.59 launches (in this case, an additional 144 candidates would be needed at preclinical phase to achieve 0.59 launches, at an additional cost of $3.3 billion).

### (vi) Sensitivity analysis

For the sensitivity analysis we adopted a method detailed by Mestre-Ferrandiz *et al.* at the United Kingdom Office of Health Economics in their study “The R&D Cost of a New Medicine” ^8^. Young *et al.* also used this method ^6^. Based on this methodology, we altered the model’s parameters for cost per phase and attrition per phase for each archetype by 10 percent higher and 10 percent lower. We also examined the impact of all possible combinations of these changes (e.g., 10% higher probability of success per phase and a 10% higher cost per phase, 10% higher probability of success per phase and a 10% lower cost per phase, etc.). We conducted this sensitivity analysis for moving current candidates through the pipeline both in our direct comparison analysis and our analysis of the complete 2019 pipeline. Additionally, we conducted this sensitivity analysis for the costs of developing priority “missing” products. Since the length of time is independent of the cost variables in P2I model, we did not conduct a sensitivity analysis varying the length of time per phase.

## Results

### (i) Candidates identified by the 2017 and the direct comparison 2019 pipelines

For the direct comparison analysis (the “apples-to-apples” approach), we only included candidates that were identified using the same method and scope used in 2017, as described in sub-section (ii) above. Using these inclusion criteria, we identified 690 candidates in the 2019 direct comparison pipeline that met the inclusion criteria for being entered into the P2I.v2 tool, an increase of 152 candidates from 2017 (see *Underlying data:* Data File 3).

Table 2 shows the change in the number of candidates from 2017 to 2019 due to changes in the pipeline. The largest increase was for diagnostics, an increase of 67 candidates from 2017 to 2019, followed by vaccines (increase of 48 candidates), biologics (increase of 19 candidates), and repurposed drugs (increase of 13 candidates). For NCEs, there was a reduction in the number of candidates (2 fewer candidates in 2019 than in 2017).

**Table 2.** Product candidates in the 2017 and 2019 direct comparison pipelines, categorized by archetype and complexity.

		Number of candidates		
Archetype	Complexity	2017	2019 Direct comparison pipeline	Difference due to changes in the pipeline
Vaccines	Simple	87	86	-1
	Complex	19	58	39
	Unprecedented	102	112	10
	**Total vaccines**	**208**	**256**	**48**
NCEs	Simple	64	40	-24
	Complex	44	66	22
	**Total NCEs**	**108**	**106**	**-2**
Diagnostics	Assay development	58	131	73
	Simple	43	37	-6
	**Total diagnostics**	**101**	**168**	**67**
Repurposed drugs	Simple	44	59	15
	Complex	46	44	-2
	**Total repurposed drugs**	**90**	**103**	**13**
Biologics	Simple	7	12	5
	Complex	8	22	14
	**Total biologics**	**15**	**34**	**19**
Other products		16	23	7
	**Total other products**	**16**	**23**	**7**
**Total**		**538**	**690**	**152**

Table 3 compares candidates between the 2017 and 2019 direct comparison pipelines by disease and indicates the change in the number of candidates from 2017 to 2019 due to changes in the pipeline. The largest increase in the number of candidates was for Ebola (an increase of 62 candidates), followed by reproductive health (28), and TB (22). The largest decreases were for HIV/AIDS (a decrease of 10 candidates), schistosomiasis (7) and non-typhoid Salmonella (3).

**Table 3.** Product candidates in the 2017 and 2019 direct comparison pipelines, categorized by disease.

Number of candidates			
Disease	2017	2019 Direct comparison pipeline	Difference due to changes in the pipeline
Buruli ulcer	4	6	2
Chagas	18	16	-2
Cholera	3	2	-1
Cryptococcal meningitis	1	3	2
Cryptosporidiasis	0	1	1
Dengue	7	9	2
Ebola	20	82	62
Enterotoxigenic E.coli (ETEC)	8	6	-2
Giardia	1	1	0
HAT (Sleeping sickness)	6	4	-2
Hepatitis C	16	15	-1
HIV/AIDS	99	89	-10
Hookworm	2	3	1
Leishmaniasis	14	19	5
Leprosy	2	2	0
Leptospirosis	1	6	5
Lymphatic filariasis	2	2	0
Malaria	109	127	18
Meningitis	2	11	9
Multiple diarrhoeal diseases	1	2	1
Multiple salmonella infections	0	1	1
Multiple vector borne diseases	1	4	3
Non-typhoidal Salmonella (NTS)	7	4	-3
Onchocerciasis	4	6	2
Pneumonia	8	12	4
Reproductive health	59	87	28
Rheumatic fever	2	4	2
Rotavirus	5	11	6
Schistosomiasis	16	9	-7
Shigellosis	13	14	1
Trachoma	2	2	0
Trichuriasis	1	1	0
Tuberculosis	98	120	22
Typhoid & paratyphoid	6	9	3
**Total**	**538**	**690**	**152**

### (ii) Candidates identified in the complete 2019 pipeline

The complete 2019 pipeline included a total of 754 product candidates that were identified in the pipeline portfolio review and met the inclusion criteria for inputting into the P2I.v2 model, representing an increase of 216 product candidates compared with the 2017 pipeline (see *Underlying data:* Data File 3). Table 4 and Table 5 show the product candidates in the 2017 and the complete 2019 pipelines, broken down by archetype ( Table 4) or by disease ( Table 5). In both tables, the difference in the number of candidates between 2017 and 2019 is further broken down into “difference due to changes in the pipeline” and “difference due to changes in scope.”

**Table 4.** Product candidates in the 2017 and the complete 2019 pipelines categorized by archetype and complexity.

		Number of candidates			Change due to	
Archetype	Complexity	2017	Complete 2019 pipeline	Difference	Pipeline expansion	Scope expansion
Vaccines	simple	87	98	11	-1	12
	Complex	19	61	42	39	3
	Unprecedented	102	112	10	10	0
	**Total vaccines**	**208**	**271**	**63**	**48**	**15**
NCEs	simple	64	49	-15	-24	9
	Complex	44	71	27	22	5
	**Total NCEs**	**108**	**120**	**12**	**-2**	**14**
Diagnostics	Assay development	58	149	91	81	10
	simple	43	30	-13	-14	1
	**Total diagnostics**	**101**	**179**	**78**	**67**	**11**
Repurposed drugs	simple	44	62	18	15	3
	complex	46	49	3	-2	5
	**Total** **repurposed** **drugs**	**90**	**111**	**21**	**13**	**8**
Biologics	simple	7	12	5	5	0
	complex	8	38	30	14	16
	**Total biologics**	**15**	**50**	**35**	**19**	**16**
Other products		16	23	7	7	0
	**Total other** **products**	**16**	**23**	**7**	**7**	**0**
**Total**		**538**	**754**	**216**	**152**	**64**

**Table 5.** Product candidates in the 2017 and the complete 2019 pipelines categorized by disease.

	Number of candidates		Changes due to	
Disease	2017	2019	Pipeline expansion	Scope expansion
Buruli ulcer	4	6	2	0
Chagas	18	16	-2	0
Chlamydia	Not in scope	5	0	5
Cholera	3	2	-1	0
Cryptococcal meningitis	1	3	2	0
Cryptosporidiasis	0	1	1	0
Dengue	7	9	2	0
Ebola	20	82	62	0
Enterotoxigenic E.coli (ETEC)	8	6	-2	0
Giardia	1	1	0	0
Gonorrhea	Not in scope	11	0	11
HAT (Sleeping sickness)	6	4	-2	0
Hepatitis B	Not in scope	8	0	8
Hepatitis C	16	15	-1	0
Herpes simplex-2	Not in scope	7	0	7
HIV/AIDS	99	105	-10	16
HPV- cervical cancer	Not in scope	1	0	1
Hookworm	2	3	1	0
Leishmaniasis	14	19	5	0
Leprosy	2	3	0	1
Leptospirosis	1	6	5	0
Lymphatic filariasis	2	2	0	0
Malaria	109	127	18	0
Meningitis	2	11	9	0
Multiple diarrhoeal diseases	1	2	1	0
Multiple diseases	0	1	0	1
Multiple salmonella infections	0	1	1	0
Multiple vector borne diseases	1	4	3	0
Mycetoma	Not in scope	1	0	1
Non-typhoidal Salmonella (NTS)	7	4	-3	0
Onchocerciasis	4	6	2	0
Pneumonia	8	12	4	0
Reproductive health	59	100	28	13
Rheumatic fever	2	4	2	0
Rotavirus	5	11	6	0
Schistosomiasis	16	9	-7	0
Shigellosis	13	14	1	0
Trachoma	2	2	0	0
Trichuriasis	1	1	0	0
Tuberculosis	98	120	22	0
Typhoid & paratyphoid	6	9	3	0
**Total**	**538**	**754**	**152**	**64**

Of the 216 additions from 2017 to 2019, 152 candidates were added from a “true” change in the pipeline and 64 product candidates were added due to a scope expansion. Out of these 64 product candidates, 31 products were identified due to either including a new archetype in the scope or due to change in the inclusion criteria of existing archetypes ( Table 6). The remaining 33 candidates identified due to the scope expansion belong to new diseases included in the 2019 pipeline. These were 5 candidates for chlamydia, 11 candidates for gonorrhea, 8 candidates for hepatitis B, 7 candidates for herpes simplex-2, and one candidate each for human papilloma virus-cervical cancer and mycetoma. These candidates are included in Table 4 under scope expansion.

**Table 6.** Additional candidates included in 2019 pipeline due to the expansion of the G-FINDER scope.

Disease	Archetype	Number of candidates
**HIV/AIDS**	Biologics	16
**Reproductive health**	New chemical entity	7
	Repurposed drugs	6
**Leprosy**	Vaccine	1
**Multi-disease**	Diagnostic	1
**Total**		**31**

Table 7 summarizes the changes in classification for the eight diagnostic product candidates based on the updated information available in 2019.

**Table 7.** Summary of the changes in classifications of eight diagnostic candidates.

Disease	Original classification (in the direct comparison pipeline)	New classification (in the complete 2019 pipeline)	Number of candidates
HIV/AIDS	Simple platform	Assay development	3
Tuberculosis	Simple platform	Assay development	2
Hepatitis C	Simple platform	Assay development	2
Malaria	Simple platform	Assay development	1

### (iii) Overview of expected product launches and costs

The 2019 direct comparison pipeline is expected to lead to 196 launches by the year 2031 at a total cost of $19.9 billion, and the complete 2019 pipeline is expected to lead to 207 launches by the year 2031 at a total cost of $21 billion ( Table 8) (see *Underlying data:* Data File 4 for the 2019 direct comparison pipeline model and Data File 5 and Data File 6 for the complete 2019 pipeline models).

**Table 8.** Summary of expected launches and costs to move candidates through 2017, direct comparison and complete pipelines of 2019.

	2017 pipeline	Direct comparison 2019 pipeline	Complete 2019 pipeline
Expected launches by 2031	128	196	207
Costs to move candidates through pipeline ($, millions)	16,348.78	19,869.98	21,027.18

### (iv) Comparison of expected launches from the 2017 pipeline versus the direct comparison 2019 pipeline

**Figure 3.**
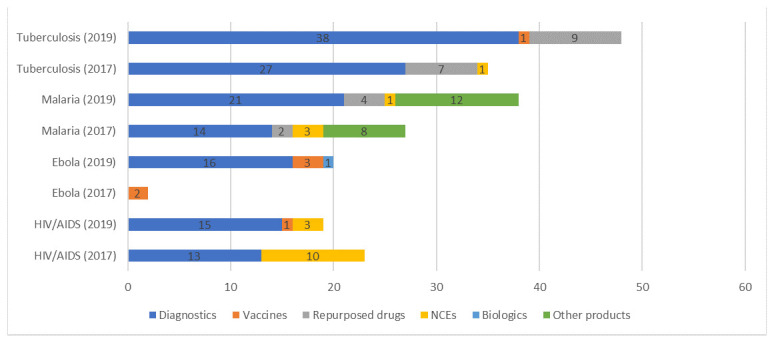
Number of expected launches for HIV/AIDS, TB, malaria and Ebola: 2017 versus the direct comparison 2019 pipelines.

The direct comparison 2019 pipeline is expected to lead to 105 combined launches of products to control HIV/AIDS, TB, and malaria; in comparison, the 2017 pipeline was expected to lead to 85 combined launches for these three diseases ( Figure 3). For all other poverty-related and neglected diseases, the direct comparison 2019 pipeline is expected to lead to 91 launches compared to 43 launches from the 2017 pipeline. Among diseases included in the direct comparison analysis, the largest increase in the number of expected launches based on the 2017 versus direct comparison 2019 pipelines would be for Ebola: the 2017 pipeline was expected to lead to two launches, and the direct comparison 2019 pipeline is expected to lead to 20 launches. This increase reflects the significant growth in the number of Ebola pipeline candidates under active development at the time of the 2019 review compared to the 2017 review.

A key finding of the direct comparison analysis is that the 2019 pipeline is expected to lead to launches of HIV and TB vaccines. In our previous study, based on the 2017 pipeline, launches of HIV and TB vaccines were *not* expected (i.e. there was a probability of launch of under 1.0 for each) ^6^. Table 9 summarizes the expected number of launches based on the 2017 and the direct comparison 2019 pipeline, broken down by disease. The difference in expected launches between the 2017 and the direct comparison 2019 pipelines are summarized under the fourth column, labeled “Expected launches: difference due to changes in the pipeline,” in Table 9.

**Table 9.** Number of expected launches from the 2017 versus the direct comparison 2019 pipelines.

	Expected launches		
Disease	2017	2019 Direct comparison pipeline	Difference due to changes in the pipeline
Buruli ulcer	2	3	1
Chagas	3	3	0
Cholera	1	0	-1
Dengue	2	3	1
Ebola	2	20	18
Enterotoxigenic E.coli (ETEC)	1	1	0
HAT (Sleeping sickness)	2	3	1
Hepatitis C	8	11	3
HIV/AIDS	23	19	-4
Leishmaniasis	3	6	3
Leprosy	0	1	1
Leptospirosis	1	6	5
Lymphatic filariasis	1	2	1
Malaria	27	38	11
Meningitis	0	5	5
Multiple vector borne diseases	0	1	1
Pneumonia	1	3	2
Reproductive health	8	14	6
Rotavirus	2	3	1
Schistosomiasis	3	2	-1
Shigellosis	2	2	0
Trachoma	0	1	1
Tuberculosis	35	48	13
Typhoid & paratyphoid	1	1	0
**Total**	**128**	**196**	**68**

### (v) Expected launches based on the complete 2019 pipeline analysis

As shown in Table 8, the complete 2019 pipeline is expected to lead to 207 launches by the year 2031 compared with 196 expected launches by the year 2031 from the direct comparison 2019 pipeline. The 11 additional launches from the complete 2019 pipeline analysis compared to the 2019 direct comparison pipeline are due to (a) the scope expansion (which led to 64 newly identified candidates, as summarized in Table 5 and Table 6), and (b) the reclassification of eight diagnostics, as summarized in Table 7. The reclassification of diagnostics increased the number of candidates classified as assay development for some diseases resulting in additional launches compared to the 2019 direct comparison pipeline. The expected launches resulting from the complete 2019 pipeline analysis, which had an expanded scope of diseases, include six additional expected launches for diseases not included in the 2017 pipeline review. These launches are one NCE for hepatitis B; three diagnostics for chlamydia; and two diagnostics for gonorrhea.

The scope expansion also added an expected launch of one biologic for HIV/AIDS and one repurposed drug for reproductive health. The remaining three additional launches were all diagnostics—for hepatitis C, TB, and malaria—and these additions are related to the changes in classification for diagnostic candidates.

The expected additional launches based on the complete 2019 pipeline analysis compared to the direct comparison 2019 pipeline are summarized under “Difference in expected launches due to: scope expansion” and “Difference in expected launches due to: classification changes” in Table 10.

**Table 10.** Expected launches by disease based on the 2017 and the complete 2019 pipelines.

	Expected launches		Difference in launches due to		
Disease	2017	Complete 2019 pipeline	Changes in the pipeline	Scope expansion	Classification changes
Buruli Ulcer	2	3	1		
Chagas	3	3	0		
Chlamydia	Not in scope	3	0	3	
Cholera	1	0	-1		
Dengue	2	3	1		
Ebola	2	20	18		
Enterotoxigenic E.coli (ETEC)	1	1	0		
Gonorrhea	Not in scope	2	0	2	
HAT (Sleeping sickness)	2	3	1		
Hepatitis B	Not in scope	1	0	1	
Hepatitis C	8	12	3		1
HIV/AIDS	23	20	-4	1	
Leishmaniasis	3	6	3		
Leprosy	0	1	1		
Leptospirosis	1	6	5		
Lymphatic filariasis	1	2	1		
Malaria	27	39	11		1
Meningitis	0	5	5		
Multiple vector borne diseases	0	1	1		
Pneumonia	1	3	2		
Reproductive health	8	15	6	1	
Rotavirus	2	3	1		
Schistosomiasis	3	2	-1		
Shigellosis	2	2	0		
Trachoma	0	1	1		
Tuberculosis	35	49	13		1
Typhoid & paratyphoid	1	1	0		
**Total**	**128**	**207**	**68**	**8**	**3**

### (vi) Estimated costs to move candidates through the pipeline based on the 2017 and the direct comparison 2019 pipelines

**Figure 4.**
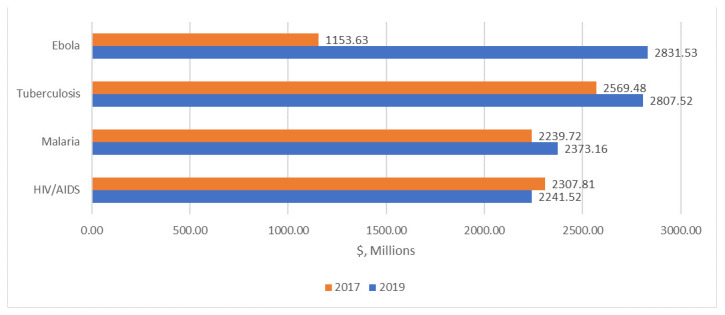
Costs from the direct comparison analysis of moving candidates for HIV/AIDS, TB, Ebola, and malaria to launch.

It would cost a total of $19.9 billion to move the candidates in the direct comparison 2019 pipeline through to launch, compared with $16.3 billion to move candidates in the 2017 pipeline to launch, an increase of $3.5 billion from 2017 to 2019. The largest cost increase for any single disease category is for Ebola: it would cost an estimated $1.2 billion to move candidates in the 2017 pipeline to launch, compared with $2.8 billion to move candidates from the 2019 pipeline to launch, an increase of $1.7 billion. Comparing the direct comparison 2019 pipeline against the 2017 pipeline as a baseline, the cost to move candidates through the pipeline to launch increased by $238 million and $133.4 million for TB and malaria, respectively, while the cost to move candidates through the pipeline fell by $66.3 million for HIV/AIDS. Figure 4 compares the costs of moving candidates for HIV/AIDS, TB, Ebola, and malaria through the pipeline to launch based on the 2017 versus the direct comparison 2019 pipelines. Table 11 compares the costs to move candidates through the pipeline based on the 2017 and direct comparison 2019, disaggregated by disease (the cost differences are summarized under the column “Cost difference due to changes in the pipeline”). 

**Table 11.** Cost to move candidates to launch by disease for the 2017 and 2019 direct comparison pipelines.

Disease	2017 ($, million)	2019 pipeline for the direct comparison analysis ($, million)	Difference due to pipeline expansion ($, million)
Buruli ulcer	51.34	65.18	13.84
Chagas'	681.25	433.92	-247.33
Cholera	324.88	219.09	-105.80
Cryptococcal meningitis	18.33	42.61	24.28
Cryptosporidiosis	0.00	18.33	18.33
Dengue	82.29	113.64	31.35
Ebola	1153.63	2831.53	1677.91
Enterotoxigenic *E.coli* (ETEC)	538.73	421.54	-117.20
Giardia	54.30	54.24	-0.05
HAT (Sleeping sickness)	74.57	31.07	-43.50
Hepatitis C	540.77	385.81	-154.96
HIV/AIDS	2307.81	2241.52	-66.30
Hookworm	147.95	197.18	49.24
Leishmaniasis	456.19	396.74	-59.45
Leprosy	18.20	17.39	-0.81
Leptospirosis	5.50	21.00	15.50
Lymphatic filariasis	18.29	7.00	-11.29
Malaria	2239.72	2373.16	133.45
Meningitis	91.32	947.94	856.62
Multiple diarrhoeal diseases	13.61	61.02	47.40
Multiple salmonella infections	0.00	37.05	37.05
Multiple vector borne diseases	73.97	91.95	17.98
Non-typhoidal Salmonella (NTS)	289.63	141.66	-147.97
Onchocerciasis	104.45	142.33	37.89
Pneumonia	644.20	1249.16	604.95
Reproductive health	908.55	1394.05	485.50
Rheumatic fever	111.00	222.03	111.02
Rotavirus	618.55	976.35	357.80
Schistosomiasis	830.37	407.38	-422.98
Shigellosis	855.13	824.27	-30.86
Trachoma	74.06	40.55	-33.51
Trichuriasis	17.60	17.58	-0.02
Tuberculosis	2569.48	2807.52	238.04
Typhoid & paratyphoid	433.10	638.20	205.09
**TOTAL**	**16348.78**	**19869.98**	**3521.19**

### (vii) Costs to move candidates through the pipeline based on the complete 2019 pipeline

Compared to the direct comparison 2019 pipeline, an additional $1.2 billion is required to move candidates in the complete 2019 pipeline through the pipeline to launch (in the narrative part of this section, the individual costs do not always add up to the totals mentioned due to rounding; Table 12 gives the individual costs). This $1.2 billion increase in cost is due to the inclusion of additional product candidates as a result of the scope expansion. The largest increase in cost was for HIV/AIDS: it would cost an estimated $2,241.5 million to move candidates in the direct comparison 2019 pipeline through to launch, compared with $2,661.9 million to move candidates in the complete 2019 pipeline to launch, an increase of $420.4 million. This increase is explained by the addition of 16 biologic candidates for HIV/AIDS control in the complete 2019 pipeline compared with the direct comparison 2019 pipeline. For reproductive health, it would cost an estimated $1,394.1 million to move candidates in the direct comparison 2019 pipeline through to launch, compared with $1,579.9 million to move candidates in the complete 2019 pipeline to launch, an increase of $185.9 million. This increase is explained by the addition of 13 candidates for reproductive health in the complete 2019 pipeline compared with the direct comparison 2019 pipeline.

**Table 12.** Cost to move candidates to launch by disease for the 2017 and complete 2019 pipelines.

	Cost ($, million)			Cost difference due to		
Disease	2017	Complete 2019 pipeline	Difference ($, million)	Changes in the pipeline ($, million)	Scope expansion ($, million)	Classification Changes ($, million)
Buruli ulcer	51.34	65.18	13.84	13.84		
Chagas'	681.25	433.92	-247.33	-247.33		
Cholera	324.88	219.09	-105.80	-105.80		
Chlamydia	Not in scope	27.02	27.02		27.02	
Cryptococcal meningitis	18.33	42.61	24.28	24.28		
Cryptosporidiosis	0.00	18.33	18.33	18.33		
Dengue	82.29	113.64	31.35	31.35		
Ebola	1153.63	2831.53	1677.91	1677.91		
Enterotoxigenic E.coli (ETEC)	538.73	421.54	-117.20	-117.20		
Giardia	54.30	54.24	-0.05	-0.05		
Gonorrhea	Not in scope	280.96	280.96		280.96	
HAT (Sleeping sickness)	74.57	31.07	-43.50	-43.50		
Hepatitis B	Not in scope	122.26	122.26		122.26	
Hepatitis C	540.77	191.76	-349.01	-154.96		-194.05
Herpes Simplex-2	Not in scope	416.58	416.58		416.58	
HIV/AIDS	2307.81	2661.89	354.07	-66.30	683.30	-262.93
Hookworm	147.95	197.18	49.24	49.24		
HPV-cervical cancer	Not in scope	5.40	5.40		5.40	
Leishmaniasis	456.19	396.74	-59.45	-59.45		
Leprosy	18.20	80.30	62.10	-0.81	62.91	
Leptospirosis	5.50	21.00	15.50	15.50		
Lymphatic filariasis	18.29	7.00	-11.29	-11.29		
Malaria	2239.72	2276.14	36.42	133.45		-97.02
Meningitis	91.32	947.94	856.62	856.62		
Multiple diarrhoeal diseases	13.61	61.02	47.40	47.40		
Multi-Disease	0.00	74.29	74.29		74.29	
Multiple salmonella infections	0.00	37.05	37.05	37.05		
Multiple vector borne diseases	73.97	91.95	17.98	17.98		
Mycetoma	Not in scope	18.58	18.58		18.58	
Non-typhoidal Salmonella (NTS)	289.63	141.66	-147.97	-147.97		
Onchocerciasis	104.45	142.33	37.89	37.89		
Pneumonia	644.20	1249.16	604.95	604.95		
Reproductive Health	908.55	1579.86	671.31	485.50	185.81	
Rheumatic fever	111.00	222.03	111.02	111.02		
Rotavirus	618.55	976.35	357.80	357.80		
Schistosomiasis	830.37	407.38	-422.98	-422.98		
Shigellosis	855.13	824.27	-30.86	-30.86		
Trachoma	74.06	40.55	-33.51	-33.51		
Trichuriasis	17.60	17.58	-0.02	-0.02		
Tuberculosis	2569.48	2641.61	72.13	238.04		-165.91
Typhoid & paratyphoid	433.10	638.20	205.09	205.09		
**TOTAL**	**16348.78**	**21027.18**	**4678.39**	**3521.19**	**1877.10**	**-719.90**

The changes in classification for the eight diagnostic candidates ( Table 7) from simple platform development in the direct comparison 2019 pipeline to assay development in the complete 2019 pipeline reduced the overall cost by $719 million. This cost reduction is due to the cost per phase being lower for assay development classification than the cost per phase for simple platform development classification for diagnostics in the P2I.v2 model.

Based on the complete 2019 pipeline analysis, Table 12 summarizes the changes in cost by disease based on the changes in the pipeline, scope expansion and classification changes. In the complete 2019 pipeline, of the total cost, 62% ($12.9 billion) would be for phase III candidates, 22% ($4.7 billion) for phase II candidates, 4% ($921 million) for phase I candidates and 12% ($2.5 billion) for pre-clinical candidates. Furthermore, in the complete 2019 pipeline, over 75% of the cost ($15.9 billion) would be incurred during the first five years.

### (viii) Results of the sensitivity analysis for moving current candidates through the pipeline

For the direct comparison 2019 pipeline, the sensitivity analysis estimated that the total cost to move the current candidates through the pipeline ranges from $16.4 billion to $23.8 billion and the expected launches range from 150 to 240 ( Table 13). For the complete 2019 pipeline, based on the sensitivity analysis results, the total cost to move the current products to launch ranges from $17.3 billion to $25.2 billion and the expected launches range from 159 to 256 ( Table 14).

**Table 13.** Sensitivity analysis for the 2019 direct comparison pipeline.

Parameters	Percent change from baseline	Estimated cost ($, millions)	Estimated number of product launches
Baseline		19,869.98	196
Probability of success	Low (-10%)	18,254.67	150
	High (+10%)	21,666.15	240
Average cost per phase	Low (-10%)	17,882.98	
	High (+10%)	21,856.98	
Combined	Low (-10% for both parameters)	16,429.20	150
	Intermediate 1 (cost +10%, probability of success -10%)	20,080.13	150
	Intermediate 2 (cost -10%, probability of success +10%)	19,499.53	240
	High (+10% for both parameters)	23,832.76	240

**Table 14.** Sensitivity analysis for the complete 2019 pipeline analysis.

Parameters	Percent change from baseline	Estimated cost ($, millions)	Estimated number of product launches
Baseline		21,027.18	207
Probability of success	Low (-10%)	19,215.54	159
	High (+10%)	23,043.73	256
Average cost per phase	Low (-10%)	18,924.46	
	High (+10%)	23,129.90	
Combined	Low (-10% for both parameters)	17,293.98	159
	Intermediate 1 (cost +10%, probability of success -10%)	21,137.09	159
	Intermediate 2 (cost -10%, probability of success +10%)	20,739.35	256
	High (+10% for both parameters)	25,238.99	256

### (ix) Estimated costs to launch missing products

Our first study using the P2I.v2 tool, based on the 2017 pipeline portfolio review, suggested that there would be 18 “missing products”, which were identified using the list of needed priority products outlined in CIH report ^6^. In the 2017 analysis, the estimated cost for launching these 18 missing products was between $13.6 billion and $21.6 billion, depending on the complexity of the product.

In the 2019 analysis, the model projects that there are likely to be launches of HIV and TB vaccines and, as a result, there would now be 16 missing products. Since the number of candidates do not change for the 16 missing product categories between the 2019 direct comparison pipeline and the complete 2019 pipeline, the missing products analysis is applicable to both pipelines. To launch one of each of the 16 key missing products, starting from pre-clinical phase, we estimate the costs to be between $5.5 billion (assuming lowest complexity products) and $14.2 billion (assuming highest complexity products). Table 15 shows the summary of these costs by disease and archetype. The ranges are calculated using the sensitivity analysis described in sub-section (vi) of the Methods.

**Table 15.** Additional number of candidates needed to achieve one launch and the costs to launch 16 missing products.

Disease	Product type	Additional no. of candidates needed at preclinical phase	Cost (US $, millions)
Tuberculosis	NCE Complex	6 (4-9)	109.9 (68.3-158.4)
Malaria	Vaccine - unprecedented	144 (98-219)	3276.7 (2141.6-5185.3)
Hepatitis C	Vaccine - simple	8 (5-12)	296.2 (216.1-371.7)
	Vaccine - complex	35 (24-53)	1068.2 (735.7-1577.2)
Multiple diarrheal diseases	Vaccine - simple	11 (8-17)	407.3 (313.3 - 538.7)
	Vaccine - complex	33 (22-50)	1007.2 (691.5-1482.5)
12 neglected tropical diseases *	NCE Simple	109 (74-164)	1490.1 (1025.1 - 2061.2)
	NCE Complex	473 (320-721)	8621.2 (5772.7-13389.9)

* These diseases are Buruli ulcer, Chagas disease, dengue, hookworm, human African trypanosomiasis (HAT), leishmaniasis, leprosy, lymphatic filariasis, onchocerciasis, schistosomiasis, trachoma and trichuriasis

For the 16 missing products, about 70% of the costs would be incurred during the first five years of product development. Therefore, the costs for the first five years range between $3.85 billion and $9.94 billion.

Over the next five years, the combined cost to move all current candidates through the pipeline and to launch a product each for 16 “missing products” ranges between $19.7 to $25.8 billion, or $3.9 to $5.2 billion per year.

## Discussion

Our study found that the product development R&D pipeline for poverty-related and neglected diseases has grown by more than a quarter in just two years, driven in particular by the global response to 2018–19 outbreaks of Ebola virus disease (EVD), as well as reproductive health, TB and malaria. As a result, the number of expected new product launches expected based on the 2019 pipeline increased by half compared to 2017, while the cost of advancing the pipeline increased by a quarter. Most of the increase in the number of pipeline candidates and the number of launches was in diagnostics, but most of the increased (and overall) costs were associated with vaccine development. The distribution of the R&D pipeline broadly aligns with the landscape of funding going to neglected diseases as assessed in the G-FINDER reports.

### Changes in the pipeline from 2017 to 2019

At August 31, 2019, there were 754 product candidates that fell within the study scope and had sufficient publicly available information to allow them to be entered into the P2I.v2 model. Of these 754 candidates, 690 were in disease and product areas that were included in our 2017 review, representing an increase of 152 candidates (or 28%) since 2017.

The pipeline growth was primarily in just four areas. Ebola, reproductive health, TB and malaria collectively accounted for 130 (86%) of the 152 additional candidates. The increase in pipeline candidates occurred for all archetypes except for NCEs, but the largest increases were in diagnostics (up by 67 candidates, a 66% increase) and vaccines (up by 48 candidates, a 23% increase), which together accounted for more than three quarters (76%) of the overall increase in product candidates.

Ebola alone was responsible for a significant proportion (41%) of the total growth of the R&D pipeline between 2017 and 2019. The number of candidates in the pipeline for Ebola increased fourfold in this period, reflecting the robust international response to two outbreaks of EVD in the Democratic Republic of the Congo (DRC) in 2018–19, the second of which – originating in North Kivu province – became the second largest EVD outbreak on record ^9^.

Although TB and malaria were among the diseases to see the most significant pipeline growth, the surge in candidates for Ebola and reproductive health helped reduce the degree to which the pipeline is dominated by HIV/AIDS, TB and malaria. While HIV/AIDS, TB and malaria still collectively accounted for close to half of all candidates in the pipeline in 2017, this proportion was down from 57% of candidates in 2017. From 2017 to 2019, the vaccine pipeline for malaria and TB was essentially stagnant, with each disease registering an increase of just a single candidate between 2017 and 2019. 

It is concerning that the size of the R&D pipeline for diarrheal diseases, salmonella infections, helminth infections and kinetoplastid infections either fell or was at best unchanged from 2017 to 2019. Of the 35 diseases included in the 2017 review, 15 still had fewer than four candidates each in the pipeline in 2019. Many of these are neglected tropical diseases, whose funding has remained basically flat over the course of the last decade, while funding for HIV/AIDS, TB, malaria and Ebola has grown significantly. Also concerning is the fact that there was no increase in the number of NCE candidates in the R&D pipeline (in fact there were two fewer in 2019 than in 2017), despite the overall pipeline growing by more than a quarter. There was a roughly even split in the number of new candidates in early-stage development and those in clinical trials or field development.

### Launches and missing products

The model suggests that the 754 candidates currently in the pipeline will deliver 207 launch-ready health technologies targeting poverty-related and neglected diseases. Looking just at the “apples-to-apples” subset, the 690 candidates in the direct comparison pipeline would be expected to deliver 196 launches, an increase of more than half (53%, an additional 68 launches) compared to the 2017 review.

Diagnostics accounted for the vast majority of the increase in expected launches in 2019 over 2017, mostly for assays rather than platforms. This increase reflected the large number of candidates entering the pipeline (especially in Ebola and TB), as well as effect of underlying assumptions of the P2I.v2 model, which applies a high probability of success for diagnostics development. More than half of the increase was for just three diseases: Ebola (up 18, 23%), TB (up 13, 37%) and malaria (up 11, 41%).

As with the pipeline distribution, expected launches are less concentrated on HIV/AIDS, TB and malaria than in the 2017 review, but again this is largely due to the surge in Ebola pipeline activity between 2017 and 2019, reflecting the coordinated global response towards unmet R&D needs to contain two back-to-back EVD outbreaks in the DRC in 2018. Based on the pipeline as of August 31, 2019, the model predicts three launch-ready Ebola vaccines. One Ebola vaccine, Merck’s Ervebo, was approved by both the European Commission and the U.S. Food and Drug Administration in late 2019. Another investigational vaccine, Janssen’s MVA-BN-Filo, is currently deployed in East and Central Africa as part of an expanded response to the DRC outbreak, and has been submitted to the European Medicines Agency for regulatory approval.

The global estimate of launch-ready HIV/AIDS products decreased from 23 in the 2017 review to 19 in the 2019 review, due to a drop in the number of HIV/AIDS NCE candidates in the pipeline. This drop reflected both the successful approval of several late-stage NCE candidates since 2017 and a lack of new candidates entering the pipeline; however, it must be noted that the HIV/AIDS therapeutics category has strict inclusion restrictions in the G-FINDER scope.

One of the most significant changes in the 2019 review compared to the 2017 review is the estimated launch of vaccines for HIV/AIDS and TB by 2031 (the 2017 pipeline would not have led to these two launches ^6^). This is a positive reflection of progress in the pipeline for these two areas since our last review, but it is worth emphasizing that the P2I model makes no assessment of the appropriateness of the projected launch-ready candidate, nor of any additional research that may be required to support product introduction. So while the model predicts the likely launch of a TB vaccine, the profile of the most advanced candidates in the pipeline suggest that – at the date of the launch projected by the model – such a vaccine may only be approved for use in limited populations. In the case of HIV/AIDS, caution may be warranted given that thus far no candidate has been able to match even the 31% efficacy achieved in the 2009 RV144 Thai Phase III clinical trials, and that during the preparation of this report one of the two most advanced HIV vaccine candidates in the pipeline (a modified version of the RV144 vaccine regimen) was found to be ineffective.

### TDR Health Product Portfolio Directory

In May 2019, TDR launched the Health Product Profile Directory (HPPD), described as an “online database describing 8–10 key characteristics (such as target population, measures of efficacy and dosage) of product profiles for medicines, vaccines, diagnostics and other products that are intended to be accessed by populations in low- and middle-income countries.” ^10^

Out of the 50 WHO-authored target product profiles (TPPs) contained in the HPPD, 28 align with the scope of this study, covering 11 diseases. Whilst not all areas with the largest pipelines have WHO-authored TPPs, most of the areas for which WHO-authored TPPs exist have healthy pipelines.

The continued expansion of the TB diagnostics pipeline (which grew by 50% between 2017 and 2019) is a case in point, but also one that highlights a potential imbalance in the current pipeline. In 2014, the WHO published a report of a consensus meeting highlighting high-priority TPPs for new TB diagnostics, divided into four use cases: (a) a sputum-based test to detect pulmonary TB; (b) a rapid biomarker-based non-sputum-based test; (c) a drug-susceptibility test; and (d) a community-based triage or referral test for identifying people suspected of having TB. In 2019, almost two-thirds (21, 64%) of all diagnostics in late-stage development for TB are rapid drug susceptibility tests; this is compared to just four rapid biomarker-based non-sputum-based test, two triage tests and a single sputum-based test to detect pulmonary TB (the remaining five late-stage candidates are not indicated for a specific TPP).

Further work to map the pipeline against the TPPs in the HPPD would provide useful insights into the nature of the R&D pipeline.

### New cost estimates

Overall, the P2I.v2 model suggests that advancing the current pipeline of 754 candidates to deliver 207 launch-ready health technologies would cost an estimated $21 billion by 2031. More than half of the total amount ($12 billion, 59%) of the cost of advancing the candidates is projected to be spent during the first three years (2020–2022), at an average cost of $3.9 billion per year.

Half of the projected costs ($10 billion, 50%) were for four diseases: Ebola ($2.8 billion, 13%), HIV/AIDS ($2.7 billion, 13%), TB ($2.6 billion, 13%) and malaria ($2.3 billion, 11%). Development of the candidates for the remaining 37 diseases each accounted for less than 10% of the total costs, most of which had less than 1% of the total share of costs.

From an archetype perspective, the main driver of the cost of development were vaccines, which accounted for almost two-thirds ($13 billion, 61%) of the total projected cost through to 2031. This was followed by diagnostics ($2.6 billion, 12%), NCEs ($2.0 billion, 9.6%), biologics ($1.9 billion, 9.3%), repurposed drugs ($1.5 billion, 7.3%) and vector control products ($79 million, 0.4%).

Looking just at the “apples-to-apples” subset, the P2I.v2 model suggests that advancing the pipeline of 690 candidates to deliver 196 launch-ready health technologies would cost an estimated $19.9 billion by 2031. This represents an increase of $3.5 billion (up 22%) over the estimate from the 2017 pipeline review.

The bulk of this $3.5 billion cost increase was due to an additional $3.1 billion in the cost of advancing vaccine candidates, and almost half (47%) was from the more than doubling of projected costs for advancing Ebola candidates (up $1.7 billion, 145%). This makes sense, as Ebola was responsible for a massive increase in the number of vaccine candidates, many of them in pre-clinical development; however the fate of these early-stage candidates is likely to be affected by the approval of Merck’s Ervebo (and pending likely approval of Janssen’s MVA-BN-Filo), as well as the waning of the current DRC outbreak.

Product development costs also increased dramatically over 2017 for meningitis (up $857 million, from a low base; although this was partly due to better sources, primarily the draft publication of the new 2030 global roadmap for meningitis vaccines) and pneumonia (up $605 million, 94%; reflecting new vaccine candidates advancing to Phase III clinical trials). Although malaria and TB account for a significant proportion of the projected costs, there was no significant increase in projected costs for these diseases between 2017 and 2019, with pipeline growth in both diseases being mainly in diagnostics rather than in other more costly archetypes.

The additional cost to launch all 16 “missing products” ranges from $5.5 billion to $14.2 billion, depending on the complexity of the products, around 70% of which would be spent in the first 5 years. Combined with the cost of advancing the current pipeline, this means that in the first 5 years the total estimated costs to move all current candidates through the pipeline and develop the 16 missing products would be around $3.9 billion to $5.2 billion per year. This total does not include funding for basic research, discovery, and post-registration studies, all of which are outside the scope of the P2I model cost projections.

How does this compare to current global funding levels? According to the most recent G-FINDER neglected disease report and two soon to be published analyses of R&D for sexual and reproductive health and emerging infectious diseases, total funding for the poverty-related and neglected diseases included in the scope of this paper totaled $3.6 billion in 2018, once funding for basic research and post-registration studies is excluded. This suggests an annual product development funding gap of at least $0.3 billion to $2.6 billion even at current record levels of neglected disease R&D funding.

### Strengths of our new study

To the best of our knowledge, this is the first study to provide time trends for the R&D pipeline of poverty-related and neglected diseases, as well as for product launches and cost estimates. Information on these trends is valuable for R&D funders, researchers, product development partnerships, and policymakers more broadly. Additional diseases were included in this new analysis including the WHO Product Development for Vaccines Advisory Committee’s priority pathogens, such as HSV-2 and gonorrhoea. 

A major strength of this study, as well as our previous study using the P2I model to examine the 2017 pipeline ^6^, is that it goes beyond analysing a single therapeutic portfolio (e.g. the pipeline of drugs for TB control ^11^) to examine the full portfolio of candidates for a very broad range of poverty-related and neglected diseases. This broader approach “aligns with the way in which funders pursue a diversified portfolio of product development projects.” ^6^ Our study helps to identify critical gaps in the global health product development pipeline by showing (a) which diseases and archetypes have few candidates, (b) which product launches are most likely, and (c) which products will probably still be missing based on existing candidates.

### Strengths of the P2I.v2 tool

We have previously described the strengths of the P2I.v2 tool in detail ^6^. In brief, we highlight three particular strengths here. First, the tool is publicly available online, as are the full details of the pipeline review, model assumptions, and model inputs and outputs. Thus readers can replicate, improve on, and further adapt our work. Second, the tool is highly flexible — users can make modifications to the underlying assumptions to see how such changes would influence the results. Third, the underlying model assumptions are likely to be realistic. These assumptions are based on a very large number of data points (e.g., assumptions on success rates and cycle times were based on data from of 25,000 development candidates), and were validated through examining peer-reviewed literature, industry reports/databases, and expert interviews ^7^. Our assumptions are roughly in line with reported industry standards (the amount of variation differs by product types) ^12, 13^.

### Limitations of our new study

The pipeline data used in this study represents a snapshot of the product development pipeline at a single point in time (up to August 31, 2019). Since the pipeline is constantly evolving, any changes—such as the advancement or termination of pipeline candidates—that occurred after this date are not reflected in the pipeline count or the resulting analysis.

Our research was limited to publicly available sources and relied on confirmation from authoritative sources, including the website of the candidate developer, recent reports from international organizations such as WHO and Unitaid, and clinical trial portals. Where we could not confirm the status of a candidate, we excluded it from the active candidate list. The lack of publicly available information on some products under development means that our count of active candidates may be an underestimate. In particular, there is comparatively less information available on pre-clinical candidates, due to the lack of dedicated public registries mirroring those available for clinical trial candidates, and limited sharing of proprietary data by companies. Our reliance on English language sources also means that the undercounting of early-stage R&D activity may disproportionately affect particular countries or regions. Early-stage candidates from the Middle East, Russia and China are likely to be underrepresented.

Our reliance on publicly available data sources also meant that we were unable to identify when each candidate had entered its current phase of development, meaning that from a model input perspective, a candidate that was close to the conclusion of its phase was treated identically to a candidate that had just entered it.

As in our 2017 study ^6^, our new study uses the consensus prioritization exercise conducted by the 2013 *Lancet* Commission on Investing in Health to identify the critical “missing” products that the current pipeline of product candidates would fail to deliver, according to the P2I.v2 model. While we believe this remains a robust global consensus, it is now seven years old. In addition, we note that the P2I. v2 model does not account for whether sufficient research and trial site capacity exists to conduct the additional studies that would be needed to deliver these missing products in the timeframe projected by the model.

### Limitations of the P2I tool

We have previously described the limitations of the P2I.v2 tool in detail ^6^. In brief, we highlight six particular limitations here.

First, the P2I tool is a simple, static, and deterministic model that does not consider (a) possible changes in the product development process, such as improved techniques, novel clinical trial designs (such as highly efficient trials ^14^), new regulatory pathways, or (b) product-related decisions, such as the decision to discontinue candidates.

Second, the tool requires users to classify every candidate based on the categories available in the model, yet it can be challenging to categorize some candidates into the P2I.v2 archetypes. In particular, there are too few archetypes for diagnostics and vector control products—for example, there are no specific archetype fields for vector control products, with both chemical and biological products included as a single archetype. 

Third, the assumptions on probabilities of success for unprecedented vaccines in phases II and III are based on a relatively small number of data points—around 10-25 data points per estimated value.

Fourth, the model assumptions used for each archetype are the same for almost every disease, with just a few exceptions (“unprecedented vaccines” refers to HIV, TB, and malaria vaccines, and there are two TB-specific archetypes, “simple NCE for TB” and “simple biologic for TB”). Therefore, the model does not take into account the specificities involved for developing a product against a specific disease. So, for example, in the model, the costs, success rates, and cycle times per phase for developing a simple vaccine for schistosomiasis would be the same as those for developing a simple vaccine for hepatitis C.

Fifth, the model does not take into account the public health value and clinical utility of a candidate, or the impact of successful product launches on the remaining candidates in the pipeline.

Finally, the model does not include discovery, early pre-clinical development and post-phase III costs such as regulatory review, marketing authorization, Phase IV and pharmacovigilance and other implementation costs. These other costs can be substantial—for example, funders have already committed $49 million to the first phase of implementation studies for the RTS,S malaria vaccine, with further funding required.

Despite these limitations, efforts are now underway, funded by TDR, to address these weaknesses and to develop further iterations of the tool. TDR is funding many different product development partnerships, including MMV, FIND, and DNDI, to use the tool to analyze their own portfolios; these analyses will also help to refine and improve the tool. TDR is also funding research institutions, such as the Graduate Institute of Geneva, to collect data that can help make further tool modifications. While we did reach out to PDPs to ask them to share new data on product development that could potentially be used to develop a version 3 of the tool (P2I.v3), the PDPs did not have large enough numbers of data points to meaningfully make tool refinements.

## Conclusions

This study has provided additional evidence that the P2I tool can be used effectively to estimate the costs and probable launches associated with moving a portfolio of current candidates for neglected diseases through the pipeline. It can help to identify gaps in the R&D pipeline and to estimate the size of the gap between funding requirements and current levels of R&D investment. Importantly, this study has also shown how the model can be used to estimate the impact of changes in the pipeline over time.

## Data availability

Open Science Framework: Analysis of the health product pipeline for poverty-related and neglected diseases using the portfolio-to-impact (p2i) modeling tool, https://doi.org/10.17605/OSF.IO/WT6VN ^15^.

This project contains the following underlying data:

- Data file 1: Full list of diseases included in 2017 and 2019 pipelines
- Data file 2: Expected launches: unrounded, rounded to nearest integer, and rounded down,
- Data file 3: Candidates in the pipeline for neglected diseases, as of August 31, 2019
- Data file 4: P2I.v2 tool showing anticipated launches and costs by disease and archetype for the 2019 direct comparison pipeline
- Data files 5 and 6: P2I.v2 tool showing anticipated launches and costs by disease and archetype for the 2019 complete pipeline
- Data files 4,5 and 6, which include the P2I modeling tool, are provided in Microsoft Excel format as the files cannot be uploaded in any other format. To use these data files please download the file and use Microsoft Excel software to open the files.

Data are available under the terms of the Creative Commons Zero "No rights reserved" data waiver (CC0 1.0 Public domain dedication).
