# Supplementary material for: Analysis of the health product pipeline for poverty-related and neglected diseases using the Portfolio-to-Impact (P2I) modeling tool
Source: F1000Res. 2020 May 21;9:416. [Version 1] doi: 10.12688/f1000research.24015.1 (PMC9120931; doi:10.12688/f1000research.24015.1)
Supplement: Peer review history

# Peer review: Reviewer response for version 1

**DOI:** 10.5256/f1000research.26491.r134990

**Published:** 2022-05-19

**Authors:** Dzintars Gotham

**Affiliations:** 1 Independent Researcher at London, London, UK; 2 King's College Hospital, London, London, UK

Overall, I do not have many or major comments. The article is very interesting, valuable, and clearly explained. It is essentially an interval update to earlier work. My congratulations to the authors.

**Comments:**

 In methods, regarding the search for candidates, the authors state that ‘government websites’ were consulted. An appendix should be provided with a precise list of sources. Was the breadth of sources the same as was used for the 2017 study? If broader, this could be added to limitations as a possible source of overestimating the 2017-2019 change.

 As the other reviewer has noted, it would be helpful to give some pointers to the reader regarding where this study’s assumed R&D costs and probability of success lie in comparison to other available estimates for per-Phase costs/POS (e.g. versus Sertkaya et al 2014, DiMasi, Prasad & Mailankody, Wouters, and versus DNDi’s reported costs of about 180 EUR million per NCE including failures – if I recall correctly).

 As the other reviewer has noticed, the authors did not apply discounting or ‘cost of capital’. In my view, this is appropriate, as there is the (implicit or explicit) assumption that the gaps in funding will/should be filled by government or philanthropic funding. In other words, COC estimation is appropriate for a business proposal, but this is not a business proposal. That said, the authors should give their reasoning behind not including COC in calculations.

 One interesting output – which I did not see in the current draft – would be an estimate of the total cost per approved product (including failures). Based on the sentence “Overall, the P2I.v2 model suggests that advancing the current pipeline of 754 candidates to deliver 207 launch-ready health technologies would cost an estimated $21 billion by 2031.” I get to a back-of-envelope estimate of 21 billion divided by 207 giving about US$101 million per launch-ready technology. Could the authors confirm whether that is correct and/or include the correct per-technology estimate? And additionally give a per-product average estimated development cost in the NCE group, in the diagnostics group, and in the vaccines group.

**Minor points:**

 The authors use the wording ‘move through pipeline’ and variations throughout the article. It was not clear to me whether this means ‘all the way’ through the pipeline i.e. to product approval. For example, in the Methods section (i), they write ‘through the pipeline from advanced preclinical to phase III clinical trials’ … does ‘to phase III clinical trials’ mean not including Ph III trials? Figure 2 does make it clear that what they mean is all the way to approval. Including the phrase ‘to approval’ in relevant places may be helpful.

 In Table 1, the authors imply that for imatinib the underlying disease pathogenesis was not understood, which is not true; imatinib was developed specifically to inhibit the BCR/ABL tyrosine kinase.

 I found this sentence confusing: “Product development costs also increased dramatically over 2017 for meningitis (up $857 million[)]and pneumonia (up $605 million[)]” - current wording implies the costs have INHERENTLY become higher for developing products for these diseases. Unless I am mistaken, the authors really mean ‘product development EXPENDITURES increased…’

Is the work clearly and accurately presented and does it cite the current literature?

Yes

If applicable, is the statistical analysis and its interpretation appropriate?

Yes

Are all the source data underlying the results available to ensure full reproducibility?

Yes

Is the study design appropriate and is the work technically sound?

Yes

Are the conclusions drawn adequately supported by the results?

Yes

Are sufficient details of methods and analysis provided to allow replication by others?

Yes

Reviewer Expertise:

Public health, health economics, health policy

I confirm that I have read this submission and believe that I have an appropriate level of expertise to confirm that it is of an acceptable scientific standard.

# Peer review: Reviewer response for version 1

**DOI:** 10.5256/f1000research.26491.r63798

**Published:** 2020-09-09

**Authors:** Mikel Berdud

**Affiliations:** 1 Office of Health Economics, London, UK

The work by Gavin Yamey and colleagues ‘Analysis of the health product pipeline for poverty-related and neglected diseases using the Portfolio-to-Impact (P2I) modelling tool’ is a relevant and original research on an important topic: the additional funding needed for poverty-related and neglected disease (PRND) product development.

 Based on a modelling exercise, the study provides evidence to answer two relevant questions:

- How much funding is needed to progress the PRND related 2019 pipeline to the market based on an analysis of the period 2019-2031.
- How large is the funding gap needed to cover to develop and produce at least one launch in each disease listed for the modelling – based on a list of a total of 45 diseases?

Methods are sound and well-described, and data are transparent and publicly available. The model is also accessible and therefore analyses can be easily replicate and scrutinised by readers.

 My first comment is that this study is a follow-up analysis of the previous work by Young *et al. *(2018) ^1^. The current analysis replicates the same estimates using the same methods but with an updated pipeline data and expanded disease list – from 35 diseases to a 45 diseases. It is important to contextualise the present work this way in order to correctly assess its contribution. In that sense, I would like to highlight which in my opinion are the two the contributions that can be drawn from the reviewed article:

  

- **The study demonstrates that PRNDs product R&D pipeline is a vivid organism** which only in two years has experienced a huge evolution. The analysis that authors call “2019 direct comparison pipeline” which is based in the same list of 35 diseases of Young *et al.* (2018) ^1^ shows that the pipeline has increased in 152 new drugs (+28%) with vaccines (+48) and diagnostics (+67) leading this increment and diseases like Ebola (+62), TB (+22) and Malaria (+18) being the most benefited. This can be considered an endogenous change of the PNRDs related pipeline. A change of this magnitude in just two-year period evidences the necessity for policymakers to track the evolution of the pipeline closely and frequently to update the expected launches and required funding to see where and by how much the focus has to be placed to encourage the most needed and impactful innovation. Learnings from continuous analysis of the pipeline may help to shape the Pharmaceutical market regulation and incentives (i.e. push and pull funding programmes).
- **The PRNDs is also by itself an evolving set of diseases.** The previous work in which this new study is based was only published two years ago. In that study a total number of 35 PNRDs were listed in 2017. Replicating criteria, a mix of clinical and socio-economic variables, the number has been increased to 45 in 2019. This shows that the changing socio-economic environment can affect the pipeline for PNRDs by “exogenously” affecting the list PNRDs. This is also an extremely relevant finding as it suggests that the optimal policy to tackle public health issues related to PNRDs requires a continuous update of what is included in the PNRDs list and which implications these inclusions may in terms of the required funding to progress the pipeline or to guarantee at least one treatment option in a reasonable time horizon.

In general, results are clear and well explained and easy to follow even for a non-expert reader, discussion is well written and goes through the most relevant findings and methodological details and the conclusion draws form results and discussion. There are many comments that I would like to provide to authors though, most of the requests for clarification and some other points that will require authors' attention and response. In order to be “indexed” i.e. listed in academic research output databases, I think authors should respond to all of them:

 Comment 1: In page 4, section (i) authors discuss assumptions on development costs, attrition rates, and cycle times.

- On development costs, there is a reference to Terry *et al. *(2018) ^2^. All development costs are well described and showed in the reference. However, I suggest to discuss and compare the cost per phase estimates used in this work with other presented in the literature. How do the used estimates compare with estimates of each development phase costs of DiMasi *et al.* (2016) ^3^, Prasad and Mailankody (2017) ^4^, and Wouters *et al. *(2020) ^5^?
- I have three questions for the assumptions on attrition rates:
  1. How are the success/attrition rates applied? How exactly do they affect the cost of R&D? is the cost of failed projects not accounted for in the calculation? This, surely, can be inferred from the P2I.v2 model which is available, but as a reader, I miss an explanation in the paper. More generally, the diagram of figure 1 does not describe in detail how the assumptions affect the cost estimate. I think the box in the middle of the diagram ‘P2I Model’ requires further visual explanation. What about something like Figure 2.4. (p. 15) of Mestre-Ferrandiz *et al. *(2012) ^6^.
  2. Are attrition rates being applied by development phase? Or are authors basically applying the overall likelihood of approval (LOA) to each archetype without phase specific discrimination? I think this is important to clarify.
  3. Have they been updated from the 2017 work? There is a new paper published in 2019 with new estimates by Wong et al. (2019) ^7^ that may be worth comparing. An interesting sensitivity may be to replicate estimates by using probabilities of success of other recently published works like Hay *et al.* (2014) ^9^ or Thomas *et al.* (2016) ^8^ or even those used by DiMasi *et al.* (2016) ^3^ and DiMasi *et al.* (2020) ^10^ in their analyses.

Comment 2: This is a question for clarification. How are the probabilities of success for unprecedented HIV, TB, and malaria vaccines calculated/estimated/assumed? There is no approved HIV vaccine yet so how can one assume a positive probability alpha and consequently estimate the minimum necessary products in the pipeline required to have at least one launched in the future? Are authors applying the archetype probability of success (PoS) which means that HIV vaccines have the same POS than vaccines for meningitis B or mRNA vaccines? This is what I understand is the approach, otherwise PoS would be equal to zero for these unprecedented vaccines. therefore, If the complex vaccine archetype PoS is taken as reference for unprecedented vaccines, should not we have a HIV vaccine approved already given the huge investment devoted to the disease? But we don’t and the analysis predicts one is expected to be launched based on the current pipeline, is not it tricky to assume? This is discussed by authors in the discussion section (page 22) where evidence that the HIV vaccine projects are highly uncertain to success and being launched is provided. In my opinion these specific cases should be subject to more detailed discussion perhaps acknowledging that it is likely that the estimated HIV vaccine will never progress to launch.

 Comment 3: In the methods section, (ii) Pipeline portfolio review, page 7, authors explain the review and cross-reference all major data sources used. This seems to be a huge work producing results by itself. As a reader, I would benefit from an explicit/descriptive summary explanation of the most relevant findings in each. It is also very useful for further work that other researchers would want to start. Also, a summary of what was used from each of the data sources would help understand the data. Which universities, companies, references, governments, and non-profit organizations were checked? Might this information be presented in a table, an annex or as another supplementary material file? More details will provide more credit to methods. Authors should at least consider and assess this possibility.   

 Comment 4: In the next paragraph of the above, authors explain a validation process leading to the inclusion of candidates. They were included only “if an authoritative source could confirm they were in active development”. As a reader I miss more details about both, the process and the authoritative sources. Which clinical trial portals were used and for which products? How was the process of correspondence interaction with product developers? How many products were (in numbers and in percentage) checked by each authoritative source? What was the percentage of testing? Were all products checked for inclusion/exclusion by these means?

 Comment 5: In the methods section, (iv) inputting the pipeline of candidates into the adapted P2i model, page 8, authors explain that “when a candidate is classified into a particular phase In the model, it is assumed it is at the beginning of the phase […]”. This approach overestimates the cost of progressing the pipeline as many of the included products can be at mid- or late point of their current development phase at the time of the inclusion hence the full phase cost should not be considered. How large is the size of this overestimation is uncertain and will depend on the distribution of the actual degree of progress within each phase of the products? Why do authors take this approach? Why don’t take the approach of assuming that all products are at the mid-point of each development phase? Some explanation and discussion of the degree of overestimation are missed.

 Comment 6: In the following paragraph of the above’s, authors recognise that they do not apply a discount rate to the cost estimates. They neither apply the rate of the cost of capital as the usual practice is in this type of estimation exercises (see DiMasi *et al.* (2016) ^3^, Prasad and Mailankody (2017) ^4^, Wouters *et al. *(2020) ^5^, Shuchmacher *et al.* (2016), Mestre-Ferrandiz *et al.* (2012) ^6^). Authors then neglect an important and well-established driver of the R&D cost: the cost of capital. An omission that is not properly discussed in the paper. Additionally, they neither simply discount costs although the timelines for cost estimation used is over a full decade and the impact of the discounting significant. This approach implies that the time of development for each phase and archetype is only relevant for the cost stream and time horizon  calculation, but it does not have any effect on the total funding required. I suggest to at least discuss this caveat in depth and in relation with what R&D cost estimation literature have been doing to this respect, which as I have said is opposite approach.

 Comment 7: In the following paragraph of the above, authors discuss on the conservative rounding approach they use to estimate number of launches. They round down all expected launches number estimates the model provides. Are all launches between 0 and 1 (if any) considered equal to 0? If the estimate is an expected output and cost, I suggest (especially for the cost estimate) to consider results with any rounding to be the base case– explicitly mentioning the integer component of the number as the minimum expected launches –  and then present results rounded down for comparison.

 Comment 8: On page 9, in the paragraph ‘The complete 2019 pipeline analysis’ authors explain that there are eight diagnostic candidate archetype that were changed between the 2019 direct comparison pipeline and complete 2019 pipeline. My opinion is that relevant assumptions applied to the different diagnostic archetypes vary significantly, this approach may result in misleading. If these changes are rationalised then comparison should be done by assuming them. Alternatively, should not be a better approach to change the archetype in the 2017 pipeline for direct comparison? Or is it equivalent?

 Comment 9: in results section, subsection ‘(vii) Costs to move candidates through the pipeline based on the complete 2019 pipeline”, page 18, authors assess the share of total cost / funding that belongs to each phase of development. For example, it is said that 62% of the cost is attributable to phase III. But language is loose as the reader may get confused. Is it 62% of the cost attributable only to projects currently in phase III? or is it the total cost of phase III attributable to all projects, those already in phase III and those that are not yet, but which they will be once they progress until to? I guess it is the second case but the dynamics should be explained, which means, the 62% also includes phase III cost of those projects still in phase I and phase II but which the P2I model predicts they will pass to phase III in the future.   

 Comment 10: In the results section, subsection ‘(viii) Results of sensitivity analysis…’ authors briefly describe the result of the sensitivity analysis and refer the reader to check these results in tables 13 and 14. I consider this discussion on the sensitivity analysis to be insufficient. Sensitivities should be discussed appropriately by parameter and also for all parameters as a whole. Assessment of how sensitive the model and the estimation to the different parameters are and why is necessary so as a reader one can get an idea of what is driving the cost and why. Authors should speculate on the potential implications of these sensitivities and the robustness of their assumptions. In addition, given the dynamic and evolving nature of pipelines and pharmaceutical R&D processes, it should be also discussed the implications of potential changes on the parameters that may likely happen during the time horizon of the estimation exercise. Such discussion will provide valuable information to assess the model's robustness of the estimations and the appropriateness of updating the estimates recurrently.

 Comment 11: In tables 13 and 14 )and related to comment 1/second bullet/point 1.), sensitivity analysis of the PoS result in an increase of the cost when PoS increases and vice versa. My concern here is whether the cost of failures of a given phase of development are included or not. For example, if for a given archetype, the pipeline is composed of 10 products and the PoS is 50%, then are the ten considered to calculate the cost of phase II of that archetype, or only the successes are considered? Can authors explain, please? I assume the cost of the ten products is included.

 Comment 12: In the discussion section, page 20, authors state that “As a result, the number of expected new product launches expected based on the 2019 pipeline increased by half compared to 2017, while the cost of advancing the pipeline increased by a quarter”. I guess the answer is in the model but the first question that comes to the reader’s mind is, how an increasing return relationship may be possible. How an increase in the input of 25% can produce an increase in the output of 50%? It’s true that we are measuring input in value and output in units, but even if this is taken into account, it is an input-output relationship that requires attention and explanation.  Assuming the probabilities of success have not changed, the other possible explanations are (i) the rounding of the expected launches (ii) biased increase of the pipeline for archetypes with higher PoS or lower cost of development. I suggest expanding the discussion on this result to explain why these non-linear relationships happens. This is also important to find out which of the archetypes may be the most efficient to fund (note we are no taking the effectiveness considerations which in a value based assessment should also be included).

 Comment 13: I discussion section, in the subsection “new cost estimates”, page 23, authors explain the additional cost to launch “missing products”. These estimates depend on the complexity of the products. It seems like the complexity has an impact of around +158% in terms of the cost increase. However, what 'complexity' means exactly in this context is not discussed, described or explained. As a reader, I miss more details here about what authors mean by complexity in this context, how they model it, and how such complexity translates to higher cost.

 Comment 14: In the discussion section, in the subsection ‘Strengths of the P2I.v2 tool’, page 23, authors state that their assumptions (i.e. success rates, costs per phase, and per archetype, duration of phase) are aligned with reported industry standards. They cite two references, Hay et al. (2014) ^9^ and Schuhmacher *et al.* (2016) ^11^. As far as I am concerned there are few newer works that have been published later that would be good to check and compare with the authors’ assumptions (see DiMasi *et al.* (2020) ^10^, Wong *et al. *(2019) ^7^, Thomas *et al.* (2016) ^8^ for probabilities of success and development times and Wouters *et al.* (2020) ^5^, Prasad and Maylankody (2017) ^4^, for research and development costs). Additionally, methods used for this work and those used by the literature are neither collated nor comparatively discussed.

 Comment 15: I'm not the right person to judge the English language as I am not a native English speaker. As a non-native English reader, my assessment is that the manuscript is very well and clearly written and it does not require any work to improve apart from the usual proofreading and QA.

Is the work clearly and accurately presented and does it cite the current literature?

Yes

If applicable, is the statistical analysis and its interpretation appropriate?

Not applicable

Are all the source data underlying the results available to ensure full reproducibility?

Yes

Is the study design appropriate and is the work technically sound?

Yes

Are the conclusions drawn adequately supported by the results?

Yes

Are sufficient details of methods and analysis provided to allow replication by others?

Yes

Reviewer Expertise:

Industrial organisation with specific background on the economics of innovation, regulation of pharmaceutical markets, incentives for R&D, pharmaceutical pricing and competition.

I confirm that I have read this submission and believe that I have an appropriate level of expertise to confirm that it is of an acceptable scientific standard.

## References

[1] : Developing new health technologies for neglected diseases: a pipeline portfolio review and cost model. *Gates Open Res* .2018;2: 10.12688/gatesopenres.12817.2 23 10.12688/gatesopenres.12817.2 30234193PMC6139384

[2] : Funding global health product R&D: the Portfolio-To-Impact Model (P2I), a new tool for modelling the impact of different research portfolios. *Gates Open Res* .2018;2: 10.12688/gatesopenres.12816.2 24 10.12688/gatesopenres.12816.2 30234194PMC6139376

[3] : Innovation in the pharmaceutical industry: New estimates of R&D costs. *J Health Econ* .2016;47: 10.1016/j.jhealeco.2016.01.012 20-33 10.1016/j.jhealeco.2016.01.012 26928437

[4] : Research and Development Spending to Bring a Single Cancer Drug to Market and Revenues After Approval. *JAMA Internal Medicine* .2017;177(11) : 10.1001/jamainternmed.2017.3601 10.1001/jamainternmed.2017.3601 PMC571027528892524

[5] : Estimated Research and Development Investment Needed to Bring a New Medicine to Market, 2009-2018. *JAMA* .2020;323(9) : 10.1001/jama.2020.1166 844-853 10.1001/jama.2020.1166 32125404PMC7054832

[6] : The R&D Cost of a New Medicine. *Office of Health Economics (OHE)* .2012;

[7] : Estimation of clinical trial success rates and related parameters. *Biostatistics* .2019;20(2) : 10.1093/biostatistics/kxx069 273-286 10.1093/biostatistics/kxx069 PMC640941829394327

[8] : Clinical development success rates 2006–2015. *BIO Industry Analysis* .2016;

[9] : Clinical development success rates for investigational drugs. *Nat Biotechnol* .2014;32(1) : 10.1038/nbt.2786 40-51 10.1038/nbt.2786 24406927

[10] : Development Times and Approval Success Rates for Drugs to Treat Infectious Diseases. *Clin Pharmacol Ther* .2020;107(2) : 10.1002/cpt.1627 324-332 10.1002/cpt.1627 31502248

[11] : A Review of the Pharmaceutical R&D Efficiency: Costs, Timelines, and Probabilities.2016; 10.1002/9783527693405.ch4 60-79 10.1002/9783527693405.ch4
